# Effect of nitric oxide synthase inhibition on the exchange of glucose and fatty acids in human skeletal muscle

**DOI:** 10.1186/1743-7075-10-43

**Published:** 2013-06-18

**Authors:** Ilkka Heinonen, Bengt Saltin, Jukka Kemppainen, Pirjo Nuutila, Juhani Knuuti, Kari Kalliokoski, Ylva Hellsten

**Affiliations:** 1 Turku PET Centre, PO Box 52, FI-20521, Turku, Finland; 2 Research Centre of Applied and Preventive Cardiovascular Medicine, University of Turku, Turku, Finland; 3 Department of Clinical Physiology and Nuclear Medicine, University of Turku, Turku, Finland; 4 Department of Medicine, Turku University Hospital, University of Turku, Turku, Finland; 5 Exercise and Sport Sciences, Section of Human Physiology, University of Copenhagen, Copenhagen, Denmark; 6 Copenhagen Muscle Research Center, University of Copenhagen, Copenhagen, Denmark

**Keywords:** Nitric oxide, Metabolism, Energy substrates, Humans

## Abstract

**Background:**

The role of nitric oxide in controlling substrate metabolism in humans is incompletely understood.

**Methods:**

The present study examined the effect of nitric oxide blockade on glucose uptake, and free fatty acid and lactate exchange in skeletal muscle of eight healthy young males. Exchange was determined by measurements of muscle perfusion by positron emission tomography and analysis of arterial and femoral venous plasma concentrations of glucose, fatty acids and lactate. The measurements were performed at rest and during exercise without (control) and with blockade of nitric oxide synthase (NOS) with *N*^G^-monomethyl-l-arginine (L-NMMA).

**Results:**

Glucose uptake at rest was 0.40 ± 0.21 μmol/100 g/min and increased to 3.71 ± 2.53 μmol/100 g/min by acute one leg low intensity exercise (p < 0.01). Prior inhibition of NOS by L-NMMA did not affect glucose uptake, at rest or during exercise (0.40 ± 0.26 and 4.74 ± 2.69 μmol/100 g/min, respectively). In the control trial, there was a small release of free fatty acids from the limb at rest (−0.05 ± 0.09 μmol/100 g/min), whereas during inhibition of NOS, there was a small uptake of fatty acids (0.04 ± 0.05 μmol/100 g/min, p < 0.05). During exercise fatty acid uptake was increased to (0.89 ± 1.07 μmol/100 g/min), and there was a non-significant trend (p = 0.10) for an increased FFA uptake with NOS inhibition 1.23 ± 1.48 μmol/100 g/min) compared to the control condition. Arterial concentrations of all substrates and exchange of lactate over the limb at rest and during exercise remained unaltered during the two conditions.

**Conclusion:**

In conclusion, inhibition of nitric oxide synthesis does not alter muscle glucose uptake during low intensity exercise, but affects free fatty acid exchange especially at rest, and may thus be involved in the modulation of energy metabolism in the human skeletal muscle.

## Introduction

The translocation of glucose transporter GLUT 4 to muscle sarcolemma appears to be the key step in mediating contraction induced glucose uptake, but the mechanisms that trigger the process are still poorly characterized [1-3]. One mediator that has been postulated to trigger GLUT 4 translocation and the subsequent increased glucose uptake is nitric oxide (NO), whose formation is increased from rest to muscle contractions [4,5]. An improved understanding of the regulation of glucose uptake, including the role of NO, in human skeletal muscle is important, especially considering that skeletal musculature accounts for ~70-80% of postprandial glucose uptake in human body [6].

NO has, in some vitro and in vivo studies in animals and humans been shown to play a role in the regulation of glucose uptake in skeletal muscle [7-13]. Moreover, exogenously applied NO donors have also been shown to enhance resting glucose uptake [10,14-16], although not all studies support this finding [17]. In terms of exercise, there is also some evidence for an effect of NOS inhibition on glucose uptake during muscle contractions in animals [7,13] and during moderate intensity whole body exercise in humans [8,11], whereas other animal studies have documented unchanged glucose uptake during exercise or electrically-induced contractions in response to acute or chronic inhibition of NO synthesis [9,10,18]. Moreover, in a human study where synthesis of NO was inhibited locally by infusion of L-NMMA into the exercising muscle via a microdialysis catheter, only local blood flow but not glucose uptake was reduced [19]. Also, in cardiac muscle, which displays a similar energy substrate utilization as skeletal muscle during low contraction intensity, it has been consistently shown in animals that NO reduces glucose uptake [12,20-23]. Hence, it is clear that the data in the literature show highly discrepant findings on the role of NO on glucose metabolism and studies especially in humans are warranted.

In a recent study with the use of positron emission tomography (PET) methodology, we reported that the inhibition of nitric oxide enhances oxygen consumption in human skeletal muscle [24], a finding that potentially could be related to an alteration in energy substrate utilization, such as altered exchange of fatty acids. In this regard, animal studies have indicated that the exchange of free fatty acids may be enhanced in response to NOS inhibition [25,26], leading also to enhanced free fatty acid oxidation as recently reviewed [27], but this aspect has not previously been investigated in humans. Thus, the aim of the present study was to determine the role for NO in glucose and fatty acid uptake in skeletal muscle by measurements of muscle specific blood flow by PET with radio-labelled water and arterial and venous concentrations of glucose and fatty acids. We hypothesised that the inhibition of NOS will reduce the uptake of glucose and enhance the exchange of free fatty acids at rest and during exercise.

## Methods

### Subjects

Eight healthy untrained young men (26 ± 2 yrs, 184 ± 4 cm, 82 ± 8 kg, 24.2 ± 1.9 kg/m^2^) volunteered to participate in this study. Central and local hemodynamic data, but not any of the substrate metabolic findings have previously been published [24]. The purpose, nature, and potential risks of the study were explained to the subjects before they gave their written informed consent to participate. None of the subjects had chronic diseases, were taking regular medication or were smokers. The study was performed approximately at four hours after the subjects had eaten their normal breakfast (approximately 450 Kcal, carbohydrates, proteins and fat contributing to 55%, 15% and 30% energy from total. The subjects abstained from caffeine-containing beverages for at least 24 h before the experiments. The subjects were also requested to avoid strenuous exercise within 48 h prior to the study. The study was performed according to the Declaration of Helsinki and was approved by the Ethical Committee of the Hospital District of South-Western Finland and National Agency for Medicine.

### Study design

Before the PET experiments, the antecubital vein was cannulated for tracer administration. For blood sampling, a radial artery cannula was placed under local anesthesia in the contralateral arm. Additionally, cannulas were placed under local anesthesia into the femoral artery and vein for local drug infusions and blood sampling, respectively. Subjects were then moved to the PET scanner with the femoral region in the gantry and the right leg was positioned in an in-house designed leg exercise dynamometer. PET measurements were first performed at resting baseline and thereafter during exercise without any drug infusion, but only during control saline. Thirty minutes later, resting and exercising measurements were performed during NOS blockade with *N*^G^-monomethyl-l-arginine (L-NMMA) (Clinalfa, Laufelfingen, Switzerland). L-NMMA was infused intra-arterially with a concentration of 1.0 mg min^−1^ kg leg mass^−1^[28]. The infusion of the drug started ten minutes before the scanning (blood flow measurement) and continued until the end of the experiments. Additionally, radial artery and femoral vein blood samples for energy substrate and blood gas parameters were drawn for analysis (7 min after the onset of steady state exercise) during each of the conditions described above. Systemic mean arterial pressure (MAP) was measured (Omron, M5-1, Omron Healthcare, Europe B.V. Hoofddorf, The Netherlands) on every occasion studied.

### Perfusion measurements and analysis

Radio water positron-emitting tracer [^15^O]-H_2_O was produced as previously described [29] and the ECAT EXACT HR + scanner (Siemens/CTI, Knoxville, TN, USA) was used in 3D mode for image acquisition to measure muscle blood flow. The oxygen-15 isotope was produced with Cyclone 3 cyclotron (IBA Molecular, Belgium). Photon attenuation was corrected by 5-min transmission scans performed at the beginning of the PET measurements performed at rest and during exercise. All data were corrected for dead time, decay and measured photon attenuation, and the images were reconstructed into a 256 × 256 matrix, producing 2.57 × 2.57 mm in-plane dimensions of voxels with 2.43 mm plane thickness. For the measurement of perfusion at rest, scanning began simultaneously with the infusion, and consisted of the following frames; 6 × 5 s, 12 × 10 s and 7 × 30 s at rest and 6 × 5 s and 12 × 10 s during exercise. During exercise, scanning was started five minutes after exercise onset to obtain a metabolic steady-state situation and continued until the end of the exercise bout, e.g. 2.5 min (7,5 min totally). Arterial blood radioactivity was also sampled continuously with a detector during imaging for perfusion quantification. Exercise consisted of dynamic m. quadriceps femoris (~2.5 kg muscle mass) one-legged exercise at 40 rpm with an average work load of 4.5 kg and with a knee angle range of motion of ~ 75–80 degrees [30]. Local muscle blood flow was measured from the m. quadriceps femoris. The data analysis was performed using the standard models [31] and methods [32,33].

### Magnetic resonance imaging

Structural Magnetic Resonance Imaging (MRI) was performed about one week before the PET study as described earlier [34], when subjects were also accustomed to the one-leg knee extension exercise model in a PET scanner. MRI scanning was performed to obtain total leg volume of the working leg since NOS inhibiting drug infusions were based on effective concentrations per litre leg volume [28]. The mean total leg volume of the subjects was 12.2 ± 1.5 l.

### Biochemical analysis

Blood samples for energy substrates (free fatty acids, glucose and lactate) and blood gases were drawn from femoral vein and radial artery in each study condition at the mid time-point of PET measurement and analysed with standardized hospital practises. Lactate and free fatty acids were analyzed with enzymatic methods (Roche Modular P analyzer, Roche Diagnostics GmbH, Mannheim, Germany) and glucose was determined in duplicates by the Glucose hexokinase method (Roche Modular P analyzer, Roche Diagnostics GmbH, Mannheim, Germany) and the average was used for concentration and following calculations. Uptake or release of energy substrates were determined by the Fick principle, thus a-v differences were multiplied with muscle blood flow.

### Statistical analysis

Statistical analyses were performed with SAS 8.2 and SAS Enterprise 4.2 programs (SAS Institute, Cary, NC). Statistical analyses were performed using two-way ANOVA for repeated measures (exercise and drug as factors). If a significant main effect(s) was found, pair wise differences were identified using the Tukey-Kramer post hoc procedure. Results are expressed as mean ± SD. A p value ≤ 0.05 was considered statistically significant.

## Results

**Figure 1:**
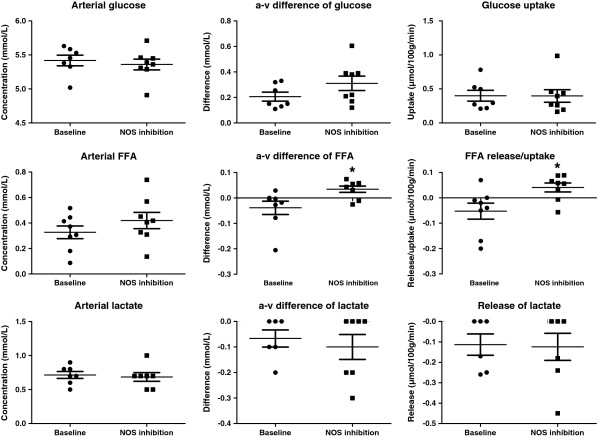
The effect of nitric oxide synthase (NOS) inhibition on arterial glucose, free fatty acids (FFA) and lactate and their arterial-to-venous (a-v) differences and uptake/release at rest. * p <0.05 compared to baseline.

**Figure 2:**
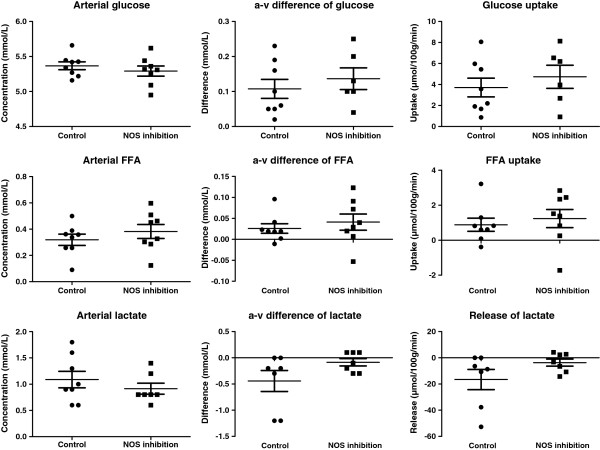
The effect of nitric oxide synthase (NOS) inhibition on arterial glucose, free fatty acids (FFA) and lactate and their arterial-to-venous (a-v) differences and uptake/release during exercise.

The arterial concentrations of glucose and FFA were not affected by inhibition either at rest (Figure 1) or during exercise (Figure 2). Glucose uptake at rest was 0.40 ± 0.21 μmol/100 g/min and increased to 3.71 ± 2.53 μmol/100 g/min by acute one leg exercise (p < 0.01) (Figure 1). Inhibition of NOS did not affect glucose uptake at rest (Figure 1) or during exercise (Figure 2), although it reduced (P < 0.05) resting muscle blood flow and increased (P < 0.05) oxygen extraction and uptake substantially (Table 1). Inhibition of NOS altered the release of free fatty acids (FFA) at rest from a release of FFAs, to an uptake (P < 0.05) during NOS blockade (Figure 1). During exercise, FFA uptake was similar during the two conditions, although there was a tendency for a higher uptake (p = 0.10) during NOS inhibition. Arterial lactate concentrations and exchange of lactate over the muscle at rest and during exercise remained unaltered during the two conditions (Figures 1 and 2). During exercise muscle blood flow and muscle oxygen extraction and consumption were similar during the control condition and NOS inhibition (Table 2).

**Table 1:** Heart rate, blood pressure, blood flow and oxygen uptake at rest

	**BASELINE**	**L-NMMA**
HR (bpm)	56 ± 6	53 ± 8
BPs (mmHg)	137 ± 15	139 ± 13
BPd (mmHg)	78 ± 10	85 ± 8*
MAP (mmHg)	98 ± 11	103 ± 9
Muscle blood flow (ml/100 g/min)	2.2 ± 0.8	1.3 ± 0.5**
Oxygen extraction (ml/L)	58 ± 18	108 ± 22***
Oxygen consumption (ml/100 g/min)	0.11 ± 0.03	0.13 ± 0.03*

*HR* heart rate, *MAP* mean arterial pressure, *BPs* systolic blood pressure, *BPd* diastolic blood pressure, * p < 0.05, ** p < 0.01 and *** p < 0.001 compared to BASELINE.

**Table 2:** Heart rate, blood pressure, blood flow and oxygen uptake during exercise

	**CONTROL**	**L-NMMA**
HR (bpm)	80 ± 11	73 ± 10
BPs (mmHg)	158 ± 23	160 ± 19
BPd (mmHg)	90 ± 12	95 ± 12
MAP (mmHg)	113 ± 15	117 ± 13
Muscle blood flow (ml/100 g/min)	36.2 ± 4.9	34.8 ± 7.9
Oxygen extraction (ml/L)	125 ± 13	132 ± 16
Oxygen consumption (ml/100 g/min)	4.50 ± 0.60	4.55 ± 0.99

*HR* heart rate, *MAP* mean arterial pressure, *BPs* systolic blood pressure, *BPd* diastolic blood pressure.

## Discussion

We report in the present study that inhibition of endogenous NO formation does not alter glucose uptake of human skeletal muscle at rest or during low intensity exercise. However, by affecting the release and uptake of free fatty acids, NO appears to contribute to the regulation of muscle energy metabolism, at least when the muscle is at rest.

### The effect of nitric oxide on muscle glucose uptake at rest and during exercise

In the present study we show that glucose uptake is unaffected by prior NOS inhibition with L-NMMA both at rest and during exercise. Previous human studies that have addressed the role of nitric oxide for glucose uptake in skeletal muscle have shown discrepant findings where some have shown that glucose uptake during exercise is reduced when NOS is inhibited [8,11], whereas others have shown no effect [19]. The discrepancy between findings in the different studies is unclear, however, differences in experimental conditions between the studies could in part explain the findings. Firstly, McConell and co-workers [8] began infusion of L-NMMA ten minutes after steady state exercise, while we began infusion ten minutes before exercise. Copp and colleagues showed that the timing of NOS inhibition does have an effect on the blood flow response in relation to muscle fibre type in rats [35], but it may also affect glucose uptake. Another experimental difference lies in the mode and intensity of exercise. In our study single leg exercise at ~10 watts with a substantial isometric component, was used whereas in the study by Bradley and co-authors the exercise consisted of two leg cycling at the 60% of peak VO_2max_, corresponding to ~142 watts [8]. Consequently, glucose uptake was increased 30-fold ~ in the study by Bradley et al. whereas we observed a ~10-fold increase in our study. The approach and results of Kingwell et al. was similar to Bradley et al. [8,11]. Thus, our present findings combined with that of others [19] suggest that glucose uptake at rest and during low-to-moderate exercise intensity is not NO mediated, but NO affects glucose uptake during higher exercise intensities [8,11]. Many previous animal studies that have addressed the effect of NO on glucose uptake in muscle have also resulted in contrasting conclusions [7,9,10,13,18], whereas most studies on cardiac muscle have all reported increased glucose uptake in parallel with increased carbohydrate metabolism during the inhibition of NOS [12,20-22]. Thus, overall, the role of NO for glucose uptake appears to be more important in cardiac than skeletal muscle.

### The effect of nitric oxide on free fatty acid exchange in muscle

In line with the results of Rottman et al. in mice [26], the present study demonstrates that NO inhibition alters the exchange of FFAs over the limb. At rest in the control condition, there was a release of FFA from the limb, whereas during NOS inhibition an uptake of FFA was detected (Figure 1). This finding, combined with the unaltered glucose uptake during NOS blockade, is also in line with the observation that oxygen consumption of the muscle is increased during NOS inhibition, and suggests that the overall metabolism, as indicated by the change in oxygen consumption, of the muscle was enhanced during NOS inhibition. The finding suggests that nitric oxide suppresses fatty acid metabolism in resting human skeletal muscle, which is in accordance with findings in vitro and animal studies showing that NOS inhibition increases FFA oxidation [27].

Whether the observed increase in FFA utilization also resulted in increased FFA oxidation is unclear as this was not determined in the present study. Nevertheless, the increase in oxygen consumption during the NOS blockade could indicate that there was an increased FFA oxidation. Many animal [36,37] and human [38] studies indicate that increased rates of FFA oxidation lowers the efficiency of the muscle. However, a switch to exclusive use of fatty acids as an energy source is calculated to impair the efficiency of ATP production by only 10–15% [39]. Our results do not suggest a complete substrate switch and, thus, the increased FFA uptake and utilization cannot fully explain the observed increase in oxygen consumption. Therefore, an additional explanation for the increase in oxygen uptake during NOS blockade probably was a reduced inhibitory influence of NO on mitochondrial respiration [40-42]. Alternatively, utilization of FFAs may have actually been enhanced secondarily to this phenomenon to fulfil increased cellular metabolism, but this possibility warrants further investigation. Finally, it has been observed that the inhibition of NOS leads to enhanced lipolysis in subcutaneous adipose tissue [43,44]. In our study arterial FFA levels appeared to be somewhat increased both at rest and during exercise, but this did not reach statistical significance, which points to the conclusion that indeed shift in the utilization rather than increased availability accounted for the observed increase in FFA uptake.

### Methodological considerations

Muscle biopsies were not obtained in the current study so direct measurements of the degree of NOS inhibition could not be obtained. The dose of L-NMMA used in the present study was similar to that used in a number of studies from our own as well as other laboratories. These previous studies, e.g. Rådegran and Saltin 1999, have shown that resting blood flow as well as the responses to acethylcoline infusions are approximately halved with use of this L-NMMA dose, indicating an effective inhibition [45]. In the current study, resting blood flow during L-NMMA infusion was reduced to a similar extent as previously observed. Moreover, it has been previously demonstrated that infusion of the NOS blocker L-NAME that reduces limb blood flow to a similar extent as the L-NMMA dose used in the current study, reduces NO synthase activity by approximately 70% [46]. Hence, it appears likely that the extent of NOS inhibition was similar in the present as in previous studies on humans [45,46]. Finally, as a vehicle control group was not applied in the present study, it is not possible to completely eliminate the possibility that there may have been a carry over effect during the second bout of exercise with NOS inhibition.

In conclusion, endogenous nitric oxide does not appear to change glucose uptake of human skeletal muscle at rest or during low intensity exercise, but shifts a release of free fatty acids to uptake, thereby altering muscle energy metabolism, in particular at rest.

## Competing interests

None of the authors had personal or financial conflict of interests.

## Authors’ contributions

All authors contributed to the conception and design of the experiments, the collection, analysis and interpretation of data, and to drafting of the article or revising it critically for important intellectual content. All authors also approved the final version of the manuscript.
